# Lebetin 2, a Snake Venom-Derived B-Type Natriuretic Peptide, Provides Immediate and Prolonged Protection against Myocardial Ischemia-Reperfusion Injury via Modulation of Post-Ischemic Inflammatory Response

**DOI:** 10.3390/toxins11090524

**Published:** 2019-09-10

**Authors:** Bochra Tourki, Anais Dumesnil, Elise Belaidi, Slim Ghrir, Diane Godin-Ribuot, Naziha Marrakchi, Vincent Richard, Paul Mulder, Erij Messadi

**Affiliations:** 1 Laboratoire des Venins et Biomolécules Thérapeutiques (LR11IPT08) et Plateforme de Physiologie et de Physiopathologie Cardiovasculaires (P2C), Institut Pasteur de Tunis, Université Tunis El Manar, 1068 Tunis, Tunisia; 2 Université Carthage Tunis, 1054 Bizerte, Tunisia; 3 Normandie Univ, UNIROUEN, Inserm U1096, FHU REMOD-VHF, 76000 Rouen, France; 4 Université Grenoble Alpes, Inserm U1042, Laboratoire HP2, 38000 Grenoble, France

**Keywords:** natriuretic peptide, myocardial infarction, ischemia-reperfusion injury, inflammation, fibrosis

## Abstract

Myocardial infarction (MI) followed by left ventricular (LV) remodeling is the most frequent cause of heart failure. Lebetin 2 (L2), a snake venom-derived natriuretic peptide, exerts cardioprotection during acute myocardial ischemia-reperfusion (IR) ex vivo. However, its effects on delayed consequences of IR injury, including post-MI inflammation and fibrosis have not been defined. Here, we determined whether a single L2 injection exerts cardioprotection in IR murine models in vivo, and whether inflammatory response to ischemic injury plays a role in L2-induced effects. We quantified infarct size (IS), fibrosis, inflammation, and both endothelial cell and cardiomyocyte densities in injured myocardium and compared these values with those induced by B-type natriuretic peptide (BNP). Both L2 and BNP reduced IS, fibrosis, and inflammatory response after IR, as evidenced by decreased leukocyte and proinflammatory M1 macrophage infiltrations in the infarcted area compared to untreated animals. However, only L2 increased anti-inflammatory M2-like macrophages. L2 also induced a higher density of endothelial cells and cardiomyocytes. Our data show that L2 has strong, acute, prolonged cardioprotective effects in post-MI that are mediated, at least in part, by the modulation of the post-ischemic inflammatory response and especially, by the enhancement of M2-like macrophages, thus reducing IR-induced necrotic and fibrotic effects.

## 1. Introduction

The gold standard of therapy for acute myocardial infarction (MI) is percutaneous intervention, with the aim of restoring blood flow to ischemic myocardium as quickly as possible. Nevertheless, reperfusion itself exacerbates myocardial injury and delays the functional recovery of the ischemic heart. This phenomenon is known as ischemia-reperfusion (IR) injury. Deleterious consequences of myocardial IR are not limited to myocytes but also include coronary endothelial cells [1]. Despite advances in mechanical and pharmacological reperfusion therapy, MI still leads to a higher risk for developing heart failure (HF), a process known as ventricular remodeling, which involves structural lesions (i.e., cardiomyocyte growth and death, inflammation, collagen matrix alterations and microvascular rarefaction) in infarcted and non-infarcted myocardium. Left ventricular (LV) remodeling is governed by complex interrelated mechanisms. Among them, post-MI inflammation plays a critical role, since it triggers wound healing and scar formation, including interstitial fibrosis, a major determinant of ventricular function impairment after MI [2]. The inflammatory response is mainly characterized by neutrophil infiltration, followed by monocyte/macrophage and lymphocyte influx in ischemic myocardium. Infiltrating monocytes express a proinflammatory (M1) phenotype, followed by a switch to a healing phenotype (M2). Both phenotypes are involved in resolution of the inflammatory process [3]. Thus, new therapies to optimize temporal and spatial regulation of the inflammatory response or a direct resolution of the imbalance between pro- and anti-inflammatory components offer interesting strategies to prevent or reverse post-MI LV dysfunction.

Several pharmacological postconditioning strategies have been used to prevent detrimental IR effects [4]. Recently, the B-type natriuretic peptide (BNP) has emerged as an important therapeutic tool in patients with HF [5]. In clinical practice, pharmacological agents that enhance the biological actions of this peptide, such as nesiritide, neprilysin inhibitors, or more recently, the angiotensin receptor antagonist-neprilysin inhibitor LCZ696, have shown potential for translational research to improve HF patient care [6,7]. However, some issues related to their efficacy, benefits, cardiovascular risks, or mechanisms of action are controversial [8,9]. In particular, although BNP’s effect on cardiac remodeling and fibrosis is established [10,11], its anti-inflammatory activity is still under debate [9,12] and its direct effect on modulation of inflammatory cell subsets during IR has not yet been demonstrated [13].

Clinical results have shown that snake venom-derived compounds, such as *Dendroaspis* natriuretic peptide (DNP), may offer superior therapeutic benefits in chronic HF [14]. This is likely due to greater potency and increased stability as compared to human family members [15,16], while displaying similar benefits in cardiac ischemia through natriuretic receptor-mediated signaling [17,18]. Recent studies focused on Lebetin 2 (L2), a 38-amino acid peptide (4 kDa) isolated from *Macrovipera lebetina* venom [19,20], that shares structural homology with natriuretic peptide (NP) family members, BNP, atrial natriuretic peptide (ANP), and DNP [20] (ranked by decreasing order of homology). Interestingly, L2 exerts cardioprotection in an IR ex vivo murine model, with additional effects compared to those of BNP under the same conditions [18]. These cardiac effects are mediated through a BNP-like mechanism of action, involving the NP receptor (NPR)/cyclic guanosine monophosphate (cGMP)-mediated pathway, downstream activation of mitochondrial K_ATP_ channels, and inhibition of mitochondrial permeability transition pore (mPTP) at the time of reperfusion [18].

In the current study, we extended the reperfusion period to investigate the effect of L2 on delayed consequences of IR, in vivo, including cardiomyocyte death, collagen matrix alterations, endothelial cell rarefaction, and post-MI inflammatory response, since these parameters are determinants for tissue healing. We focused particularly on L2/BNP-induced inflammatory-cell modulation by examining M1/M2 macrophage recruitment in the infarcted heart. L2 proved effective against MI with acute and prolonged effects, after a single injection administered prior to the onset of reperfusion. To the best of our knowledge, this report describes novel insights into mechanisms of NPs in myocardial repair, since L2, but not BNP, induced an increase in M2-macrophage subtype after MI, contributing to the resolution of the inflammatory process, and subsequently reducing IR-induced necrotic and fibrotic effects.

## 2. Results

### 2.1. L2 Effect on Blood Pressure and Heart Rate

**Figure 1:**
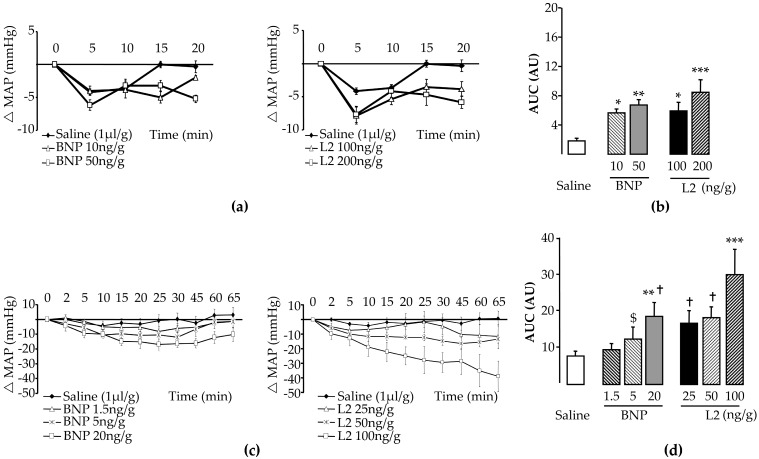
Effects of Lebetin 2 (L2) and B-type natriuretic peptide (BNP) on blood pressure. (**a**) Dose-dependent hypotensive effects of BNP (10 or 50 ng/g) and L2 (100 or 200 ng/g) in rats; (**b**) AUCs in rats, absolute decrease in MAP × time; (**c**) dose-dependent hypotensive effects of BNP (1.5, 5 or 20 ng/g) and L2 (25, 50 or 100 ng/g) in mice; (**d**) AUCs in mice, absolute decrease in MAP × time. Data are mean ± SEM. For the number of animals, see Table 1. *, *p* < 0.05, **, *p* < 0.01, ***, *p* < 0.001 vs. saline (control) group, $, *p* < 0.05 vs. BNP (20 ng/g), †, *p* < 0.05 vs. L2 (100 ng/g). △MAP, variation in mean arterial blood pressure.

To define effective doses of L2 and BNP, we investigated their influence on blood pressure and heart rate (HR, see Materials and Methods). Mean baseline values for blood pressure and HR did not differ statistically among experimental groups in either rats or mice (Table 1). BNP or L2 induced a dose-dependent decrease in the mean arterial pressure (MAP, Figure 1a,c, Table 1). The maximal hypotensive response to BNP or L2 was further documented by comparing areas under curves (AUCs, Figure 1b,d). The HR was not statistically different among experimental groups before or after treatment (Table 1). In rats, the effect of 100 ng/g L2 was equivalent to the effect of BNP at 50 ng/g (Figure 1a,b, AUCs NS). In mice, 25 ng/g L2 was equivalent to 20 ng/g BNP at inducing hypotensive response (Figure 1c,d, AUCs NS). The doses selected significantly decreased blood pressure; however, the maximal hypotensive responses to these doses, occurring within 30 min after bolus injection, were les than 30% in all cases (Figure 1a,c). Therefore, these doses were used in subsequent IR experiments, based on their ability to elicit a mild decrease in blood pressure, which minimized the deleterious effect of hypotension.

### 2.2. L2 Decreases LV Infarct Size Following IR Injury

**Figure 2:**
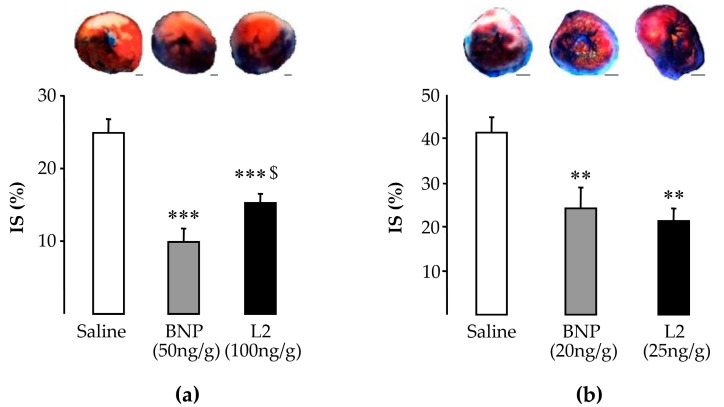
Effects of Lebetin 2 (L2) and B-type natriuretic peptide (BNP) on acute infarct size. Myocardial infarction was induced by 30-min ischemia followed by 120-min reperfusion. (**a**) Rats received saline (1 µL/g), BNP (50 ng/g) or L2 (100 ng/g) intraperitoneally, 5 min before reperfusion. (**b**) Mice received saline (1 µL/g), BNP (20 ng/g) or L2 (25 ng/g) intravenously, 5 min before reperfusion. Infarct size (IS) was measured relative to area at risk (IS/AR%) after 2,3,5-Triphenyltetrazolium chloride (TTC) staining. Images above histograms represent heart sections corresponding to experimental groups. Areas stained with Evans blue dye correspond to the tissue that had not undergone ischemia-reperfusion (IR). Red and white areas were obtained after TTC staining and correspond, respectively, to tissue that remained viable after IR and to the infarcted zone. Data are mean ± SEM. For the numbers of animals, see Table 1. **, *p* < 0.01, ***, *p* < 0.001 vs. saline (control) group, $, *p* < 0.05 vs. BNP corresponding group. Scale bars = 1 mm.

After IR, the area at risk (AR) did not differ statistically among experimental groups (Table 1). After 120 min of reperfusion, infarct size (IS) in controls averaged 24.9% ± 1.8% in rats (Figure 2a) and 41.3% ± 3.5% in mice (Figure 2b). At 100 ng/g in rats or 25 ng/g in mice, L2 significantly reduced IS/AR to 15.3% ± 1.2% (–39%, *p* < 0.001) and 21.3% ± 2.8% (–48%, *p* < 0.01) respectively (Figure 2a,b). The BNP IS-limiting effect was about 60% in rats (50 ng/g, 9.9% ± 1.9%, *p* < 0.001; Figure 2a) and 41% in mice (20 ng/g, 24.3% ± 4.7%, *p* < 0.01; Figure 2b).

**Figure 3:**
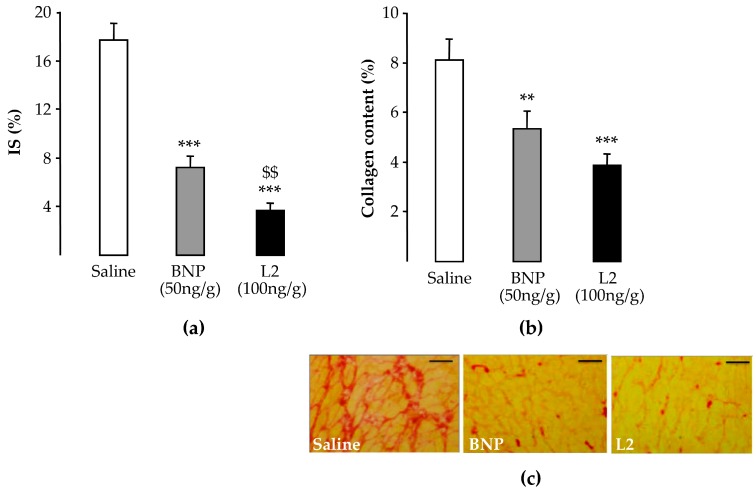
Effects of Lebetin 2 (L2) and B-type natriuretic peptide (BNP) on infarct size and fibrosis. Myocardial infarction was induced in rats by 35 min of ischemia followed by a 14-day reperfusion period. Saline (1 µL/g), BNP (50 ng/g) or L2 (100 ng/g) was administered intraperitoneally, 5 min before reperfusion. (**a**) Infarct size (IS) determined after Sirius red staining was expressed as a percentage ((endocardial + epicardial circumference of the infarcted tissue)/(endocardial+epicardial circumference of the LV) × 100); (**b**) density of collagen I and III used as a marker of fibrosis, was calculated after Sirius red staining as the area occupied by collagen/the surface of the image. On each section, several fields were photographed (15–20 images/rat) and the mean collagen density was calculated; (**c**) representative microphotographs of collagen content in Sirius red-stained myocardial slides from control and treated animals. Data are mean ± SEM. For the numbers of animals, see Table 1. **, *p* < 0.01, ***, *p* < 0.001 vs. saline (control) corresponding group, $$, *p* < 0.01 vs. BNP corresponding group. Scale bars = 50 µm, ×40.

At day 14 post-reperfusion, the L2 (100 ng/g) cardioprotective effect was maintained in rats. IS fell to 3.7% ± 0.7% [–80%, vs. control non-treated animals (17.7% ± 1.4%), *p* < 0.001; Figure 3a]. This effect was more marked than that obtained with BNP at 50 ng/g (7.2% ± 1.0%, *p* < 0.01; Figure 3a).

### 2.3. L2 Exerts Post-Ischemic Anti-Fibrotic Effect

At day 14 post-reperfusion, interstitial fibrosis, corresponding to collagen accumulation relative to the total LV area, averaged 8.1% ± 0.9% in control animals (Figure 3b). Treatments with L2 (100 ng/g) and BNP (50 ng/g) decreased fibrosis by 52% (3.9% ± 0.5%, *p* < 0.001) and 34% (5.3% ± 0.7%, *p* < 0.01) respectively, compared to the control group (Figure 3b,c).

### 2.4. L2 Modulates the Post-Ischemic Inflammatory Response

**Figure 4:**
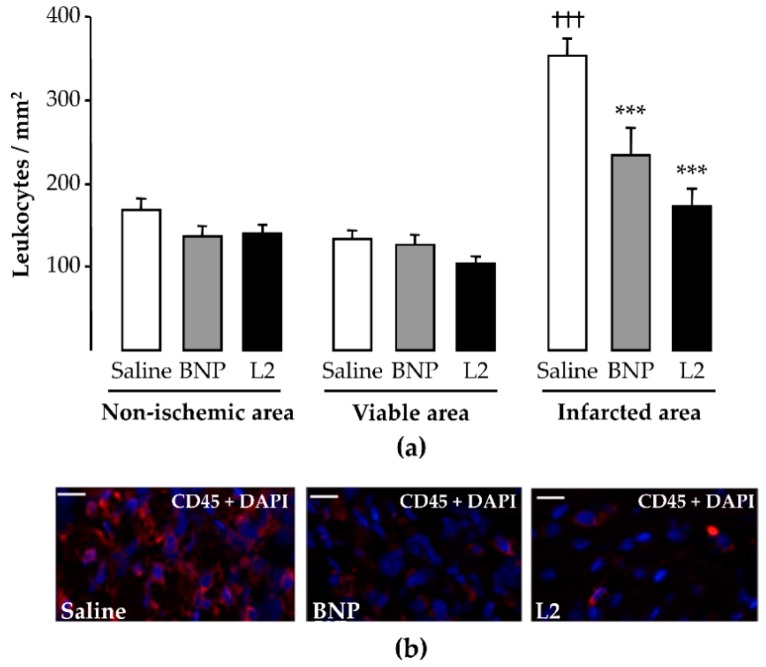
Effects of Lebetin 2 (L2) and B-type natriuretic peptide (BNP) on post-ischemic leukocyte infiltration. Myocardial infarction was induced by 35 min of ischemia followed by a 2-day reperfusion period. (**a**) Infiltrated leukocytes were assessed by CD45 immunolabeling in the infarcted area and its border zone (viable area), as well as in the septum (non-ischemic area) of rats treated with saline (1 µL/g), BNP (50 ng/g) or L2 (100 ng/g) intraperitoneally, 5 min before reperfusion. On each section, several fields were photographed (12–30 images/rat, *n* = 5–6) and the mean number of leukocytes per field was calculated; (**b**) representative microphotographs showing variable density of the leukocytes (CD45 in red and nuclei in blue) in the infarcted area from control and treated animals. Data are mean ± SEM. ***, *p* < 0.001 vs. saline (control) corresponding group, †††, *p* < 0.001 vs. corresponding other LV areas. Scale bars = 50 µm. ×20. DAPI, 4′, 6′-diamidino-2-phenylindole.

In control (untreated) rats, the number of CD45-positive cells was significantly enhanced in the infarcted LV area (353 ± 21 cells/mm^2^) compared to the viable LV area (133 ± 11 cells/mm^2^, *p* < 0.001; Figure 4a). Both L2 (100 ng/g) and BNP (50 ng/g) treatments decreased leukocyte infiltration in the infarcted area by 51% (173 ± 21 cells/mm^2^, *p* < 0.001) and 34% (234 ± 33 cells/mm^2^, *p* < 0.001), respectively, compared to controls (Figure 4a,b).

**Figure 5:**
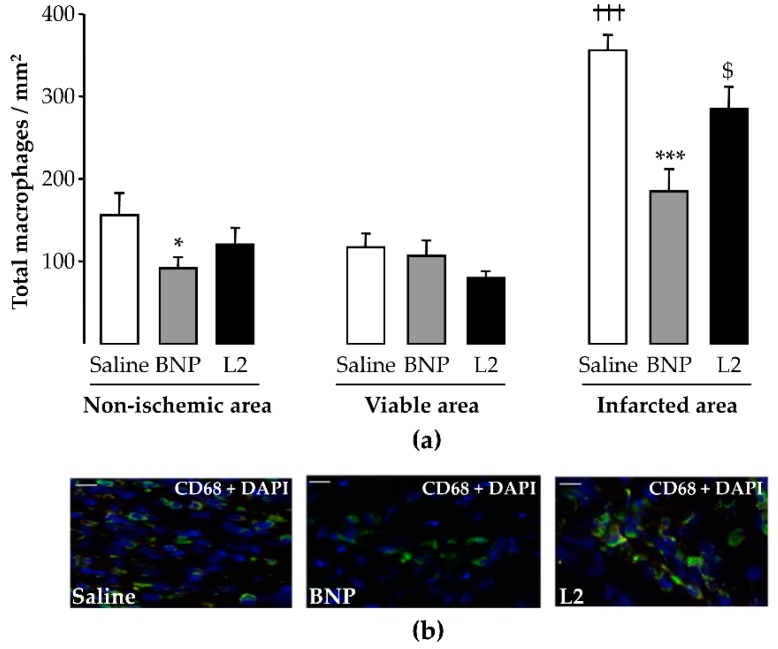
Effects of Lebetin 2 (L2) and B-type natriuretic peptide (BNP) on post-ischemic macrophage recruitment. Myocardial infarction was induced by 35 min of ischemia followed by a 2-day reperfusion period. (**a**) Infiltrated macrophages were assessed by CD68 immunolabeling in the infarcted area and its border zone (viable area), as well as in the septum (non-ischemic area) of rats treated with saline (1 µL/g), BNP (50 ng/g) or L2 (100 ng/g) intraperitoneally, 5 min before reperfusion. On each section, several fields were photographed (12–30 images/rat, *n* = 5–6) and the mean number of macrophages per field was calculated; (**b**) representative microphotographs showing the variable density of macrophages (CD68 in green and nuclei in blue) in the infarcted area from control and treated animals. Data are mean ± SEM. *, *p* < 0.05, ***, *p* < 0.001 vs. saline (control) corresponding group, $, *p* < 0.05 vs. BNP corresponding group, †††, *p* < 0.001 vs. corresponding other LV areas. Scale bars = 50 µm. ×20. DAPI, 4′, 6′-diamidino-2-phenylindole.

As with leukocytes, the number of CD68-positive cells was significantly increased in the infarcted LV area (357 ± 18 cells/mm^2^) compared to the viable LV area (117 ± 17 cells/mm^2^, *p* < 0.001; Figure 5a). Both L2 (284 ± 27 cells/mm^2^, *p* < 0.05) and BNP (185 ± 26 cells/mm^2^, *p* < 0.001) decreased the number of macrophages in the infarcted LV area by 20% and 48% respectively, compared to the control group (Figure 5a,b).

**Figure 6:**
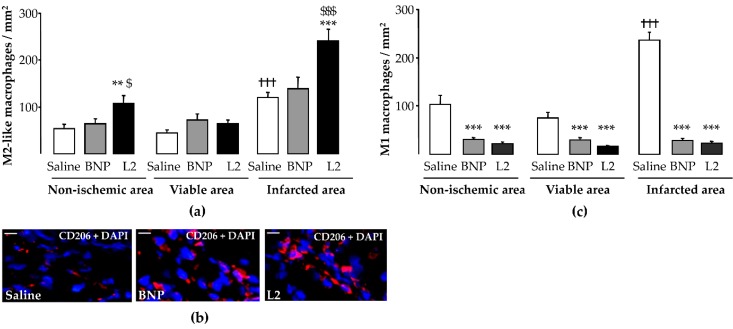
Effects of Lebetin 2 (L2) and B-type natriuretic peptide (BNP) on macrophage polarization and on recruitment of M2-like macrophages. Myocardial infarction was induced by 35 min of ischemia followed by a 2-day reperfusion period. (**a**) Infiltrated M2-like macrophages were assessed by double immunolabeling of CD68 and CD206/MRC-1 in the infarcted area and its border zone (viable area), as well as in the septum (non-ischemic area) of rats treated with saline (1 µL/g), BNP (50 ng/g) or L2 (100 ng/g) intraperitoneally, 5 min before reperfusion. On each section, several fields were photographed (12–30 images/rat, *n* = 5–6) and the mean number of M2-like macrophages per field was calculated; (**b**) representative microphotographs showing the variable density of M2-like macrophages (CD206/MRC-1 in red and nuclei in blue) in the infarcted area from control and treated animals; (**c**) proinflammatory M1 macrophages were identified as CD68^+^ or CD206^-^ cells in different LV areas of control and treated animals. Data are mean ± SEM. **, *p* < 0.01, ***, *p* < 0.001 vs. saline (control) corresponding group, $, *p* < 0.05, $$$, *p* < 0.001 vs. BNP corresponding group, †††, *p* < 0.001 vs. corresponding other LV areas. Scale bars = 50 µm. ×20. DAPI, 4′, 6′-diamidino-2-phenylindole.

To detect total versus alternatively activated macrophages, M2 macrophage polarization was determined by counting CD68 and MRC-1 double-labeled cells. For both M1 (236 ± 16 cells/mm^2^) and M2 (120 ± 11 cells/mm^2^) macrophages counted in control rats, a significant increase was observed in the infarcted LV area compared to the viable LV area (*p* < 0.001; Figure 6a,c). L2 (23 ± 4 cells/mm^2^) and BNP (28 ± 5 cells/mm^2^) decreased M1 infiltration in all LV areas with a sustained reduction in the LV infarcted area (−90% and −80% respectively, *p* < 0.001 vs. control group; Figure 6c). However, only L2 (242 ± 25 cells/mm^2^, *p* < 0.001), but not BNP (139 ± 25 cells/mm^2^), increased the number of M2-like macrophages in the infarcted area compared to control animals (Figure 6a,b).

### 2.5. L2 Enhances Endothelial Cell and Cardiomyocyte Densities upon MI

**Figure 7:**
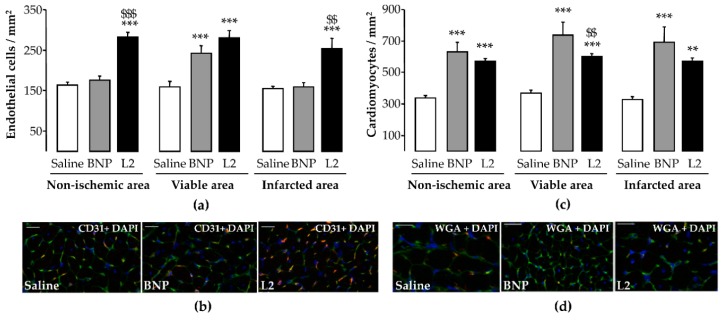
Effects of Lebetin 2 (L2) and B-type natriuretic peptide (BNP) on post-ischemic endothelial cell and cardiomyocyte densities. Myocardial infarction was induced by 35 min of ischemia followed by a 2-day reperfusion period. (**a**) Endothelial cell density was assessed by CD31 immunolabeling in the infarcted area and its border zone (viable area), as well as in the septum (non-ischemic area) of rats treated with saline (1 µL/g), BNP (50 ng/g) or L2 (100 ng/g) intraperitoneally, 5 min before reperfusion. On each section, several fields were photographed (12–30 images/rat, *n* = 5–6) and the mean number of endothelial cells per field was calculated; (**b**) representative microphotographs showing the variable density of endothelial cells (CD31 in red and nuclei in blue) in the infracted area from control and treated animals; (**c**) cardiomyocyte cell density was assessed with wheat germ agglutinin (WGA) immunolabeling in the infarcted area and its border zone (viable area), as well as in the septum (non-ischemic area) of rats treated with saline (1 µL/g), BNP (50 ng/g) or L2 (100 ng/g) intraperitoneally, 5 min before reperfusion. On each section, several fields were photographed (12–30 images/rat, *n* = 5–6) and the mean number of cardiomyocytes per field was calculated; (**d**) representative microphotographs showing the variable density of the cardiomyocytes (WGA in green and nuclei in blue) in the infarcted area from control and treated animals. **, *p* < 0.01, ***, *p* < 0.001 vs. saline (control) corresponding group, $$, *p* < 0.01, $$$, *p* < 0.001 vs. BNP corresponding group. Scale bars = 50 µm. ×40. DAPI, 4′, 6′-diamidino-2-phenylindole.

L2 treatment induced an increase in endothelial cell density after IR in all LV areas, with an increase of 64% in the infarcted area (254 ± 26 cells/mm^2^, *p* < 0.001), while BNP (160 ± 10 cells/mm^2^) failed to induce any change in endothelial cell density in this area compared to non-treated rats (155 ± 6 cells/mm^2^) (Figure 7a,b). However, cardiomyocyte density was significantly increased by both L2 and BNP in all LV areas, with an increase of 75% (568 ± 23 cells/mm^2^, *p* < 0.01) and 113% (691 ± 98 cells/mm^2^, *p* < 0.001), respectively, in the infarcted area, compared to control group (325 ± 21 cells/mm^2^) (Figure 7c,d).

## 3. Discussion

Previous (pre-)clinical studies have confirmed the beneficial effects of NPs on myocardial repair [5,11], but the underlying mechanisms of action are still poorly understood. Anti-inflammatory pathways, in particular, have not been fully investigated, and so far, no report has addressed the role of these peptides in regulating infiltrating leukocytes and macrophage subtypes after MI. We show here that L2 has the ability to repair heart damage, as evidenced by attenuation of both necrosis and IS, reduction of fibrosis, and promotion of endothelial-cell regeneration. L2 also significantly reduced leukocyte recruitment, while increasing M2-macrophage recruitment in infarcted myocardium. While other NPs required sustained administration to achieve cardioprotection [21,22,23], we show here that a single injection of L2 prior to reperfusion efficiently improved acute IR injury, with prolonged effects on days 2 and 14 post-reperfusion, highlighting the efficacy of L2 in IR, in vivo, and confirming our previous ex vivo data [18]. Our study provides novel insights into mechanisms of NPs in myocardial repair.

NPs exert vasodilating, natriuretic, and diuretic actions [24] as well as inhibitory effects on sympathetic nervous and renin-angiotensin-aldosterone systems [25], leading to a decrease in pre- and after-load. Thus, in our work, L2 exerts acute beneficial effects by maintaining the blood supply to cardiac tissue. Blood pressure-lowering effect is unlikely to account for cardioprotection in our study, since L2 and BNP were used at doses inducing only a mild, transient hypotensive effect; with no effect on HR. This is consistent with previous studies dissociating cardioprotective and blood pressure effects of vasoactive compounds in this model [26,27]. The L2 IS-limiting effect was still observed on day 14 post-reperfusion, and interestingly, was more pronounced than after acute IR, which suggests that L2, beside its effects on necrosis, likely induces other regulatory actions, the effects of which could not have been observed under acute conditions. Significantly, this was further confirmed by the reduction of post-ischemic fibrosis upon L2 treatment, as evidenced by the inhibition of collagen synthesis following prolonged reperfusion [28]. This anti-fibrotic effect may also be related to the inhibition of cardiac fibroblast growth [29] or the activation of matrix metalloproteinases [30], as reported for BNP, which accelerated infarct healing.

After MI, many inflammatory cells infiltrate the infarcted heart to convert necrotic tissue into scar tissue. However, enhanced inflammatory cell infiltration into the myocardium may exacerbate cardiac injury and worsen post-MI remodeling [29,30]. Macrophages are essential mediators of the inflammatory response, with an important role in the initiation and resolution of inflammation. It has been supposed that the healing of infarcted myocardium occurs through macrophage transition, a conversion from a pro-inflammatory (M1) phenotype to an anti-inflammatory (M2) phenotype [29]. This suggests that the modulation of the inflammatory response may improve healing and alleviate LV remodeling of the injured heart [30]. On day 2 post-reperfusion, L2 and BNP significantly reduced both leukocyte and macrophage infiltration into the infarcted area, where inflammatory cells are prominent [31,32]. Previous work has reported an association between the cardioprotective action of BNP and its inhibitory effect on proinflammatory cytokines and related mechanisms [11,13]. However, caution has been raised about transgenic mouse experiments by Kawakami et al. [12] and Izumi et al. [9], where the BNP/NPR-A axis was shown to stimulate polymorphonuclear infiltration during ischemic injury [9,12]. In the first case, a possible explanation could be that the excessive expression of BNP may be harmful, since NP-induced cardioprotection is effective at low-dose administration [33]. For the second study [9], the cardioprotection observed in NPR-A deficient mice, which was linked to a decrease of infiltrating neutrophils, may be due to compensatory mechanisms following loss of dominant receptor function [34]. Particularly, NPR-A downregulation was positively associated with increased expression of NPR-B protein in rat hearts [34], which alternatively could ensure cardioprotection [35].

Inflammatory-cell modulation by NPs has not been demonstrated yet. Therefore, our finding that the M2 macrophage population increased following L2 treatment was quite surprising. This was particularly interesting since BNP, although effective in reducing the M1 inflammatory subset, failed to induce M2 polarization. Expansion of the M2-macrophage population probably explains the increased level of total macrophages following L2 treatment (Figure 5). Altogether, our data explain the greater efficacy of L2, since M2 enrichment was associated with cardiac regeneration after MI [36]. Furthermore, many studies have reported the essential role of M2 macrophages in repair of MI and attenuation of post-MI remodeling [37,38].

IR triggers endothelial and coronary microvascular inflammation, leading to the impairment of NO-mediated, endothelium-dependent vasodilation [1]. NPs mitigate MI-induced endothelial dysfunction and subsequent release of NO [23], thereby increasing the coronary flow and blood supply to injured myocardial cells. However, despite the anti-apoptotic action of BNP [39], controversies still exist regarding the effect of the NP/cGMP signaling system on endothelial cell regeneration after vascular damage, with claims ranging from enhancement to protection [28,40]. In the present study, only the venom peptide, but not BNP, enhanced the endothelial cell number in the infarcted area, while both molecules similarly increased the number of cardiomyocytes at this site. This effect of L2 could be mediated by M2-like macrophage signaling, since both promote tissue repair and healing, including capillary network formation and proliferation capacity of endothelial cells [41]. This hypothesis is strengthened by the fact that in our work, BNP, which does not promote M2-like macrophage polarization, does not increase endothelial cell density either. This suggests that BNP in that context was not able to preserve endothelial cell viability or to induce their proliferation in injured myocardium. These results accord with previous work showing that only L2, and not BNP, efficiently increased the coronary flow [18], suggesting opposite pharmacologies of the two peptides on endothelial function. It would therefore appear that unlike BNP, L2 enhances the integrity or viability of endothelial cells. Mechanistically, these discrepancies could be attributed to differences in the affinities of L2 and BNP to natriuretic receptors. The low affinity of BNP for NP receptor type C (NPR-C) [42], which triggers PI3K/Akt/eNOS signaling [21,22,23], may provide an explanation for why BNP does not have any effect on endothelial cell function, post-MI. More mechanistic investigations are required to further explore the potential affinity of L2 to NPR-C. Such affinity has been reported for other snake venom NPs [16].

Although our results provide novel insights into mechanisms of myocardial repair triggered by NPs, they leave some unanswered questions. First, our study was primarily designed to determine whether prolonged post-MI effects of L2 and BNP are associated with the modulation of inflammatory cell response. However, we did not explore how NPs reduce leukocyte recruitment and induce the switch to M2 macrophages. Previous studies [43] reported complex mechanisms involved in leukocyte recruitment and M1-to-M2 macrophage transformation, but how NPs trigger these events specifically is unclear and awaits further investigation. Decreased leukocyte and inflammatory macrophage infiltration could be achieved through BNP-mediated inhibition of interleukin (IL)-1, IL-6 and tumor necrosis factor alpha (TNFα) secretion [44], which, in turn, could inhibit chemokine signaling via macrophage-chemoattractant protein-1 (MCP-1) and macrophage inflammatory protein 1 (MIP-1), known for their role in promoting proinflammatory macrophage infiltration [45]. On the other hand, L2 treatment may increase M2 macrophages by increasing IL-10 release, which mediates the switch from proinflammatory macrophage infiltration to alternatively activated anti-inflammatory macrophages [36], as previously reported for NPs [46]. Second, L2 may improve the survival and recruitment of M2 macrophages in the infarcted heart; however, it is still unclear whether and how the increased number of M2 macrophages actually contributes to myocardial repair.

## 4. Conclusions

The present study shows that L2 potently alleviates acute, prolonged heart damage by attenuating both post-ischemic necrosis and fibrosis. L2 also promotes endothelial-cell regeneration in infarcted myocardium and significantly reduces inflammatory cell recruitment, while enhancing M2-macrophage post-MI. These findings provide novel insights into mechanisms of NPs in myocardial repair, since many of these effects have not been observed with BNP treatment. Despite a single systemic administration of L2 at the time of reperfusion, the L2 acute, protective effects persisted up to 14 days post-MI. This can be explained by the activation of the NPR/cGMP pathway, which is known to induce acute effects such as vasorelaxation/vasodilation, but also chronic effects such as anti-fibrotic action during cardiac injury [47]. Moreover, as inflammation triggers interstitial fibrosis [48,49], it is likely that L2 provides prolonged actions (inhibition of fibrosis) as a consequence of its inhibitory effect on inflammation.

Given that L2 limits leukocyte trafficking, inhibits non-resolving inflammatory cells, and enhances pro-resolving cells, these findings are clinically relevant, as the repair response can be subjected to pharmacological interventions directed toward regressing inflammation and subsequent post-MI fibrosis. Modulation of the inflammatory response by NP therapy will be of great significance in enhancing the treatment of HF, especially in patients with intense and prolonged inflammatory responses.

## 5. Materials and Methods

### 5.1. Drugs Used in the Study

**Figure 8:**
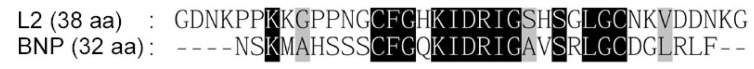
Homology between the amino acid sequences of Lebetin 2 (L2, 38 amino acids) and B-type natriuretic peptide (BNP, 32 amino acids). Identical amino acid positions are shaded in black while conserved or similar residues are in gray. L2 and BNP display 54% similarity. Alignment was performed using GeneDoc, version 2.7.000.

All experiments were performed with synthetic L2 (isoform α, 38 amino acids) purchased from Genosphère Biotechnologies (Paris, France), the production of which is based on native L2 (4 kDa), from the venom of *Macrovipera lebetina*. Prior to use of synthetic L2, we confirmed that both synthetic and native L2 exerted similar effects on the cardiac NPR/cGMP signaling and cardiodynamics of isolated rat hearts [18]. BNP (human recombinant peptide, 32 amino acids) was purchased from Sigma–Aldrich (Saint Quentin Fallavier, France). L2 and BNP share 54% structural homology based on their amino acid sequences (Figure 8).

### 5.2. Animals

Male Wistar rats (280–300 g) and male C57BL6 mice (20–30 g) were purchased from Janvier Labs (Le Genest-Saint-Isle, France). They were housed in climate-controlled conditions and had unrestricted access to standard rat chow and drinking water. Animal experiments were performed in accordance with the Guide for the Care and Use of Laboratory Animals of the National Institutes of Health (NIH Pub. No.85–23, Revised 1996), the Council of the European Communities (86/609/EEC; November 24th 1986), the French National Legislation (Ethical Approval No.76–114/ 01181.01) and also by the Biomedical Ethics Committee of the Pasteur Institute in Tunis (Ethical Approval No. 2015/08/I/LR11IPT08/V0; 9 November 2015).

### 5.3. In Vivo Dose-Response Effect on Blood Pressure

Anesthetized (60 mg/kg sodium pentobarbital, by intraperitoneal route) animals were placed on a thermally controlled heating pad (37 ± 1 °C). A catheter was inserted into the right carotid artery for blood pressure and HR recording (Iox2 software, Emka Technologies, Paris, France). Effective doses of L2 and BNP were chosen for their ability to induce mild hypotension, such as is usually observed with other vasocative compounds in IR studies [26,27], to minimize the effect of blood pressure decrease on IR outcomes. The L2 dose range was chosen based on a previous study showing that the equivalent active dose of L2 was higher than that of BNP [18]. Based thereon, blood pressure changes triggered by BNP or L2 were measured in rats treated with increasing doses of BNP (10 or 50 ng/g, *n* = 6), L2 (100 or 200 ng/g, *n* = 7) or saline (NaCl 9‰, *n* = 7). Blood pressure response was also assessed in mice treated with BNP (1.5, 5 or 20 ng/g, *n* = 5–10), L2 (25, 50 or 100 ng/g, *n* = 4–5) or saline (NaCl 9‰, *n* = 11). Drugs were injected as a single intravenous bolus (1 μL/g body weight).

The blood pressure was recorded, and the MAP was calculated at intervals after injection. AUCs (MAP absolute variations × time) were calculated in each animal according to the trapezoidal rule and averaged within each experimental group.

### 5.4. In Vivo Murine Models of Myocardial Ischemia-Reperfusion

#### 5.4.1. Surgical Preparation

For acute IR studies, animals were anesthetized with sodium pentobarbital (60 mg/kg, by intraperitoneal route). For prolonged IR studies, rats were anesthetized intraperitoneally with a mix of ketamine (50 mg/kg) and xylazine (3.6 mg/kg). They were ventilated and a thoracotomy was performed. Body temperature was monitored with a rectal probe connected to a digital thermometer, and maintained at 37 °C using a heating pad. An electrocardiogram (ECG) was recorded throughout experiments (Iox2 software, Emka Technologies, Paris, France). The left main coronary artery was occluded close to its origin using reversible ligation, as previously described [31,50]. At the end of ischemia, the coronary blood flow was restored and reperfusion achieved by loosening the suture. The lungs were then re-inflated by increasing positive end expiratory pressure, and the chest was closed during the reperfusion period.

For acute IR studies, animals were kept on the heating pad throughout the experiment and hearts were immediately excised at the end of reperfusion to assess IS. Animals submitted to prolonged reperfusion, were returned to their cages (2–3 rats/cage) after recover on a heating pad for later assessment of post-ischemic inflammatory response, IS, and fibrosis.

#### 5.4.2. Experimental Protocols

**Figure 9:**
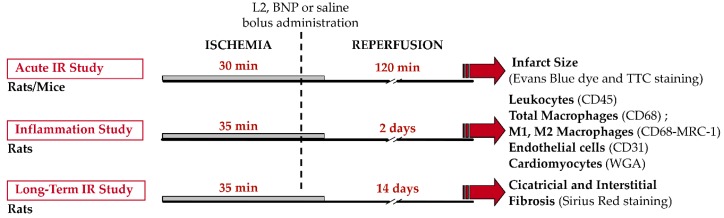
Experimental design. Three protocols of myocardial ischemia-reperfusion (IR) were applied. For acute infarct size study (upper panel), rats and mice underwent 30 min ischemia (full solid line) followed by 120 min coronary reperfusion (thin solid line). In two other additional series of experiments, rats underwent 35 min of ischemia followed by either 2 days (middle panel) or 14 days (lower panel) of reperfusion, to study respectively post-ischemic inflammation and fibrosis. Rats and mice received either B-type natriuretic peptide (BNP, 50 ng/g and 20 ng/g respectively), Lebetin 2 (L2, 100 ng/g and 25 ng/g respectively) or saline (NaCl 9‰, controls), as a single systemic bolus (1 µL/g body weight), 5 min before starting reperfusion.

Animals underwent either acute or prolonged reperfusion (Figure 9). In the first set of experiments, in an attempt to validate acute beneficial effects of L2 that have been previously demonstrated only ex vivo [18], we subjected either rats (*n* = 5–7) or mice (*n* = 5–6) to acute IR injury induced by 30 min of ischemia followed by 120 min of reperfusion (Figure 9) and assessed IS. In a second set of experiments, once the acute effect was demonstrated, we subjected rats to coronary ischemia for 35 min followed by either 2 days of reperfusion to assess cellular inflammatory response, endothelial cells, and cardiac myocytes (*n* = 5–6), or 14 days of reperfusion, to evaluate IS and fibrosis (*n* = 7–10) (Figure 9).

Rats and mice received either BNP (50 ng/g and 20 ng/g; respectively), L2 (100 ng/g and 25 ng/g; respectively) or saline (control group; NaCl 9‰). Drugs were given as a single systemic bolus (1 μL/g body weight), 5 min before reperfusion. The intravenous route was selected for drug administration in acute experiments in mice, whereas intraperitoneal injection was performed in rats.

### 5.5. Histological Analysis

#### 5.5.1. Acute Infarct Size

Acute IS was determined after 120 min of reperfusion, as described previously [31,50]. Briefly the chest was reopened at the end of reperfusion and the coronary artery was reoccluded. IS, expressed as a percentage of AR, was quantified on transverse LV slices after staining with Evans Blue and buffered 2,3,5-triphenyltetrazolium chloride (TTC) solutions. AR and IS were quantified using a computerized planimetric technique (ImageJ software, NIH, Bethesda, Maryland, MD, USA) by an observer blinded to treatment groups [31,50].

#### 5.5.2. Cardiac Morphometry: Infarct Size and Fibrosis

At day 14 post-reperfusion, rats were euthanized with a lethal dose of sodium methohexital. Hearts were rapidly excised and arrested in ice-cold saturated potassium chloride buffer, and LV conserved in Bouin’s fixative solution, as described previously [51]. After fixation, the LV was cut into several slices perpendicular to the apex-to-base axis. LV sections were dehydrated and embedded in paraffin. Then, 10-μm histological slices were stained with Sirius red for IS and fibrosis assessment.

For IS, LV sections were photographed under a light microscope (×1.25, Carl Zeiss, GmbH, Jena, Germany). Endocardial and epicardial circumferences of infarcted tissue and of the LV were determined and IS was calculated as (endocardial + epicardial circumference of the infarcted tissue)/(endocardial+epicardial circumference of the LV) × 100 using Image Pro Plus 6.3 software (Media Cybernetics, Rockville, Maryland, MD, USA) [51].

For fibrosis, cardiac collagen density was measured. Sections were photographed (×40, Carl Zeiss, GmbH, Jena, Germany), analyzed (Image Pro Plus 6.3 software, Media Cybernetics, Rockville, Maryland MD, USA) and collagen density was calculated as the surface occupied by collagen/the surface of the image [51]. On each section, several fields were photographed (15–20 images/rat) and mean collagen density was calculated.

### 5.6. Immunohistochemistry

#### 5.6.1. Post-Ischemic Inflammation

At day 2 post-reperfusion, rats were euthanized with a lethal dose of sodium methohexital to assess leukocyte and macrophage infiltration, these being maximal at approximately 48 h reperfusion time [31,32]. Hearts were rapidly excised and arrested in ice-cold saturated potassium chloride buffer, frozen in liquid nitrogen, and stored at −80 °C pending study of post-ischemic inflammatory response. Cryosections (10 µm) were post-fixed in acetone and stained according to standard protocols [52]. Leukocyte infiltration was detected using rat anti-mouse CD45 antibody (1:200; Santa Cruz, Dallas, TX, USA) reacting against most leukocytes. For the detection of total vs. M2-like macrophages, mouse anti-rat CD68 (1:200; Serotec, Oxford, UK) was used in combination with rabbit anti-mouse CD206/MRC-1 (1:300; Santa Cruz, Dallas, TX, USA). Proinflammatory M1 were identified as CD68^+^ and CD206^−^ cells. Primary antibodies were revealed using FITC-conjugated donkey anti-mouse IgG (1:300; Jackson Immunoresearch Laboratories, Inc., West Grove, PA, USA) or Cy3-conjugated donkey anti-rabbit IgG (1:300; Jackson Immunoresearch Laboratories, Inc., West Grove, PA, USA). Nuclei were stained with Vectashield mounting-medium with DAPI (Vector Laboratories, Inc., Burlingame, CA, USA) in all rat heart cryosections. Micrographs were captured using a fluorescence microscope equipped with an Apotome (×20, AxioImager Z1, Carl Zeiss GmbH, Jena, Germany) and analyzed using Image Pro Plus 6.3 software (Media Cybernetics, Rockville, MD, USA) by an operator blinded to treatment groups. Inflammatory cells were quantified as cell number per mm^2^ in various LV sections [infarcted area and its border zone (viable area) as well as in the septum (non-ischemic area)]. Several fields per section were photographed and the mean inflammatory cell number was calculated.

#### 5.6.2. Endothelial Cell and Cardiomyocyte Densities

To assess cardiac endothelial cell and myocyte densities at day 2 post-reperfusion, LV cryosections (10 µm) fixed in acetone were stained according to standard protocols [53] using rat anti-mouse CD31 (PECAM-1, 1:100; BD Biosciences-Pharmingen, San Diego, CA, USA) for the detection of blood vessel endothelial cells and wheat germ agglutinin (1:100; WGA-A488, Invitrogen, Courtaboeuf, France) for the detection of cardiomyocytes. Secondary reagents included Streptavidin–Cy3 (1:1500; Jackson Immunoresearch Laboratories, Inc., West Grove, PA, USA). Nuclei were stained with Vectashield mounting-medium with DAPI (Vector Laboratories, Inc., Burlingame, CA, USA) in all rat heart cryosections. Micrographs were captured using a fluorescence microscope (×40, AxioImager Z1, Carl Zeiss GmbH, Jena, Germany) and analyzed by an operator blinded to treatment groups using Image Pro Plus 6.3 software (Media Cybernetics, Rockville, MD, USA). Endothelial cell and cardiomyocyte densities were quantified as the number of CD31 labeled cells per mm^2^ and transversally sectioned cardiomyocytes per mm^2^, respectively in various LV sections [infarcted area and its border zone (viable area), as well as in the septum (non-ischemic area)]. Several fields per section were photographed and the mean endothelial cell or cardiomyocyte number was calculated.

### 5.7. Statistical Analysis

All results are presented as means ± S.E.M. AUCs (MAP absolute variations × time), IS, and collagen density were compared by one-way analysis of variance (ANOVA). Two-way (Treatment × LV area) repeated measure ANOVA was performed to analyze inflammation, endothelial cells, and cardiomyocytes. Differences in parameters obtained at each time point were evaluated using a Student’s *t* test or Dunnett’s post-hoc test in case of significance, using JMP software (JMP, SAS Institute Inc., Cary, NC, USA). Values of *p* < 0.05 were considered statistically significant.

## Figures and Tables

**Table 1:** Effects of B-type natriuretic peptide (BNP) and Lebetin 2 (L2) on blood pressure, heart rate, and post-ischemic areas at risk.

	*Hemodynamic Study*					*IR Study*			
Group		MAP (mmHg)		HR (bpm)		AR/LV (%)			
	*n*	Before	After	Before	After	*n*	R, 120 min	*n*	R, 14 days
*Rats*									
Control (saline 1 µL/g)	7	108 ± 5	96 ± 2	321 ± 6	310 ± 7	5	65.3 ± 3.0	10	82.4 ± 1.8
BNP (10 ng/g)	6	105 ± 7	92 ± 4	323 ± 6	315 ± 6				
BNP (50 ng/g)	6	104 ± 6	59 ± 6 *	335 ± 5	326 ± 6	5	66.6 ± 3.2	6	85.6 ± 1.3
L2 (100 ng/g)	7	105 ± 5	63 ± 3 *	326 ± 6	317 ± 6	6	66.4 ± 3.5	7	84.8 ± 1.1
L2 (200 ng/g)	7	106 ± 5	34 ± 7 *	330 ± 7	320 ± 4				
*Mice*							R, 120 min		
Control (saline 1 µL/g)	11	72 ± 3	70 ± 4	447 ± 27	422 ± 16	6	25.8 ± 2.5		
BNP (1.5 ng/g)	10	70 ± 3	62 ± 2 *	436 ± 14	467 ± 21				
BNP (5 ng/g)	5	67 ± 2	57 ± 3 *	432 ± 32	415 ± 36				
BNP (20 ng/g)	7	71 ± 6	54 ± 5 *	469 ± 35	473 ± 32	5	31.7 ± 1.2		
L2 (25 ng/g)	5	75 ± 4	64 ± 3	518 ± 9	414 ± 21	5	31.0 ± 2.6		
L2 (50 ng/g)	4	79 ± 4	63 ± 3 *	497 ± 42	412 ± 19				
L2 (100 ng/g)	5	69 ± 1	36 ± 8 *	511 ± 19	454 ± 7				

Data are mean ± SEM. For the hemodynamic study, blood pressure [mean arterial pressure (MAP)] and heart rate (HR) were recorded before and after treatment at maximal effect following BNP or L2 injection in each protocol. For the acute ischemia-reperfusion (IR) study, the area at risk (AR) was determined after 30 min of ischemia followed by 120 min of reperfusion in rats and mice. For the prolonged IR study, AR was determined after 35 min of ischemia followed by a 14–day reperfusion period in rats. bpm, beats per minute; R, reperfusion. *, *p* < 0.05 vs. corresponding value before treatment.

## Abbreviations

**AR**	Area at Risk
**AUC**	Area under curve
**BNP**	B-type natriuretic peptide
**cGMP**	Cyclic guanosine monophosphate
**DNP**	*Dendroaspis* natriuretic peptide
**HF**	Heart failure
**HR**	Heart rate
**IR**	Ischemia-reperfusion
**IS**	Infarct size
**L2**	Lebetin 2
**LV**	Left ventricle
**MAP**	Mean arterial blood pressure
**MI**	Myocardial infarction
**NO**	Nitric oxide
**NP**	Natriuretic peptide
**NPR**	Natriuretic peptide receptor
